# Cost of hospitalization for childbirth in India: how equitable it is in the post-NRHM era?

**DOI:** 10.1186/s13104-017-2729-z

**Published:** 2017-08-15

**Authors:** Jaya Prasad Tripathy, Hemant D. Shewade, Sanskruti Mishra, A. M. V. Kumar, A. D. Harries

**Affiliations:** 1 International Union Against Tuberculosis and Lung Disease (The Union), South-East Asia Regional Office, C-6, Qutub Institutional Area, New Delhi, 110016 India; 2 Independent Public Health Consultant, New Delhi, India; 3 International Union Against Tuberculosis and Lung Disease (The Union), Paris, France; 4 London School of Hygiene and Tropical Medicine, London, UK

**Keywords:** Catastrophic expenditure, Childbirth, Health care costs, Delivery

## Abstract

**Background and objective:**

Information on out-of-pocket (OOP) expenditure during childbirth in public and private health facilities in India is needed to make rational decisions for improving affordability to maternal care services. We undertook this study to evaluate the OOP expenditure due to hospitalization from childbirth and its impact on households.

**Methods:**

This is a secondary data analysis of a nationwide household survey by the National Sample Survey Organization in 2014. The survey reported health service utilization and health care related expenditure by income quintiles and type of health facility. The recall period for hospitalization expenditure was 365 days. OOP expenditure amounting to more than 10% of annual consumption expenditure was termed as catastrophic.

**Results:**

Median expenditure per episode of hospitalisation due to childbirth was US$54. The expenditure incurred was about six times higher among the richest quintile compared to the poorest quintile. Median private sector OOP hospitalization expenditure was nearly nine times higher than in the public sector. Hospitalization in a private sector facility leads to a significantly higher prevalence of catastrophic expenditure than hospitalization in a public sector (60% vs. 7%). Indirect cost (43%) constituted the largest share in the total expenditure in public sector hospitalizations. Urban residence, poor wealth quintile, residing in eastern and southern regions of India and delivery in private hospital were significantly associated with catastrophic expenditure.

**Conclusions:**

We strongly recommend cash transfer schemes with effective pro-poor targeting to reduce the impact of catastrophic expenditure. Strengthening of public health facilities is required along with private sector regulation.

## Background

India accounts for 17% of global maternal deaths [1]. The maternal mortality ratio in India showed a decline from 301 deaths per 100,000 live births in the period 2001–03 to 178 during 2010–12, but still lagged behind the Millennium Development Goal target of 109 by the year 2015 [2]. The progress in improving maternal health, as envisaged in the sustainable development goals (SDGs), critically depends on the availability, affordability and effective use of reproductive health services [3]. Health care cost is a key determinant of the utilization of maternal services [4]. In Bihar, one of India’s poorest states with high proportion of home deliveries, approximately 50% of women reported financial concerns as the reason for not availing institutional delivery care [5].

To improve the availability and accessibility to quality health care services in public health centres, the Government of India launched the National Rural Health Mission (NRHM) in 2005—the largest flagship programme in the country. Janani Suraksha Yojana (JSY) is a safe motherhood intervention under the NRHM being implemented with the objective of promoting institutional delivery by providing cash assistance to the poorest and the marginalized. In order to improve financial access to institutional deliveries, the Government of India also launched Janani Shishu Suraksha Karyakram (JSSK) in 2011 which provides free delivery and medical treatment of sick neonates for up to 30 days of birth in public health facilities. The NRHM has succeeded in injecting new energy into India’s public health system through huge expansion of infrastructure and human resources. The mission has led to enhanced utilization of public health facilities for childbirths and accelerated reduction in infant and neonatal mortality [6].

In India, total health expenditure constituted 4.25% of gross domestic product (GDP), with private and public sectors accounting for 78 and 20% respectively in 2005. OOP expenditure accounted for 70% of the total health expenditure [7]. Peters et al. estimated that a quarter of the Indian population fell into poverty as a direct result of the medical expenses incurred through hospitalisation [8].

A number of studies have explored maternal healthcare expenditure in South Asia including India, Pakistan, Bangladesh and Nepal [9–15]. However, most of these studies had two major limitations. First, they were based on small samples in localized geographical areas. Second, healthcare expenditure was calculated without reference to the household expenditure. Thus, the impact of maternal health care expenditure on households was not studied. A few studies in India such as those by Bonu et al. and Mohanty et al. have estimated out-of-pocket (OOP) maternal health care expenditure and its impact on households in large nationally representative samples.[13, 14]. But, these findings refer to pre-NRHM era as they have analysed datasets from national surveys which were conducted before or immediately after NRHM was launched. There is no recent data in the post-NRHM era to study the impact of cash incentive schemes such as JSY and JSSK which were introduced to improve financial access to institutional deliveries. Furthermore, Bonu et al. collected aggregate data for medical expenditure which may underestimate the level of expenditure, in comparison with the collection of information disaggregated by each cost item.

Information on OOP during child birth in public and private health facilities in India is needed to make rational decisions for improving affordability of health care services. There is paucity of such data from India in the post-NRHM era. We, therefore, undertook this study to evaluate the cost of maternity services, both direct and indirect for women who delivered in the last 1 year from the time of the survey which took place in 2014.

## Methods

### Aims

The objectives of the study are: (i) estimate the median OOP expenditure and prevalence of catastrophic expenditure due to hospitalization from childbirth by type of health facility and by income quintiles; and (ii) examine the inter-state variations in OOP expenditure due to childbirth in public and private health care centres across India.

### Study design

This was a secondary data analysis of a nationwide survey data collected by the National Sample Survey Organisation (NSSO), India.

### Data source

The source of data was the representative nationwide survey data collected by the National Sample Survey Organization (NSSO) in its 71st round (2014) on ‘Health’ and ‘Education’. NSSO is a national organisation under the Ministry of Statistics In India, established in 1950 to regularly conduct surveys and provide useful statistics in the field of socio-economic status of households, demography, health, industries, agriculture, consumer expenditure, etc. Results of NSSO surveys are brought out in the form of NSS reports available at the website of the Ministry at http://www.mospi.nic.in. This survey (71st round) was carried out for 6 months from January to June 2014. A stratified two-stage sampling design was adopted with sampling of census villages in the rural areas and urban frame survey blocks in the urban sector in the first stage, followed by sampling of households in the second stage. A total of 4577 villages and 3720 urban blocks were surveyed from which 36,480 and 29,452 households were sampled in rural and urban areas respectively. A total of 333,104 persons were interviewed from 65,932 households. The detailed methodology can be found in the survey report [18]. Data on maternal health care expenditure were collected for women aged 15–49 years who delivered in a hospital during 365 days prior to the survey.

The study population comprised of women aged 15–49 years who delivered in a hospital, public or private within 1 year of the survey which took place in 2014. OOP expenditure for each episode of hospitalization due to childbirth was recorded. Detailed expenditure was available for drugs; diagnostic tests (including ECG, X-ray, pathological tests, etc.); professional fees for doctors; payments to hospital/institution; other medical expenses (physiotherapy, personal medical appliances, blood, oxygen, attendant charges, etc.); and indirect costs. Indirect costs included transport for patients and other accompanying persons, food related expenses, lodging charges, etc. Household consumption expenditure was recorded as well as other socio-demographic characteristics including caste, occupation, gender and education. Data were also collected on type of facility (public or private) accessed for medical care.

The recall period for assessing household consumption expenditure was 1 month. OOP expenditure per hospitalization episode amounting to more than 10% of annual consumption expenditure was termed as ‘catastrophic’ [19, 20].

### Data analysis

Data was imported into SPSS version 17.0 for analysis. The unit of analysis was one episode of hospitalization. The study population was divided into quintile groups based on monthly per capita consumption expenditure (MPCE). Median values/percentages for all indicators were compared across five MPCE quintiles and type of health facility (public and private). Median hospitalization expenditure per episode was estimated for those who reported hospitalization due to childbirth/delivery. Inter-state variations in utilization of public health facilities and maternal health care expenditure were also reported. Since this was a multistage stratified random survey, estimates were derived by applying sampling weights given by the NSSO.

### Ethical approval

The Ethics Advisory Group of International Union Against Tuberculosis and Lung Disease (The Union), Paris, France waived the need for ethical clearance for this study (Reference Number 121/16). This is a secondary data analysis hence no consent to participate was needed.

## Results

Out of a total of 57,456 hospitalizations, 14,587 (25%) were related to childbirth, either normal vaginal delivery or caesarean section, of which 9336 (64%) were in the public sector. A total of 14,303 subjects reported 14,587 episodes of hospitalizations due to childbirth in the previous 365 days. About 98% of the respondents reported one episode of hospitalization whereas only 2% reported two episodes of hospitalization in the last year. The mean age of the respondents was 26 years (sd = 5). Nearly 86% reported paying (direct costs only) for maternal health care services.

The household monthly per capita consumption expenditure limits (in USD) for the five quintiles are as follows: first quintile (3–11), second quintile (12–24), third quintile (25–35), fourth quintile (36–52) and the fifth quintile (53–578).

### Hospitalisation-related expenditure

Median expenditure per episode of hospitalisation due to childbirth was US$54. The expenditure incurred was about six times higher among the richest quintile compared to the poorest quintile.

Median OOP hospitalization expenditure was nearly nine times higher in private sector as compared to public sector. Hospitalization in a private sector (60%) facility led to a significantly higher prevalence of catastrophic expenditure than hospitalization in a public sector (7%). Medicines accounted for 25% of public and 18% of private sector OOP hospitalization expenditure. Indirect costs (43%) constituted the largest share in the total expenditure in public sector hospitalizations. The median duration of hospitalization was 3 and 5 days in the public and private sector respectively. (Table 1).

**Table 1:** Out-of-pocket expenditure and other parameters from hospitalization due to childbirth across wealth quintiles, caste and place of residence in public and private health facilities across India

Wealth quintiles	Public	Private	Overall
1st MPCE	23 (10–44)	166 (98–282)	28 (11–67)
2nd MPCE	25 (12–52)	186 (105–346)	42 (15–113)
3rd MPCE	28 (13–58)	200 (111–337)	61 (20–154)
4th MPCE	28 (14–60)	235 (130–369)	78 (25–223)
5th MPCE	31 (14–70)	312 (156–514)	175 (54–405)
Overall	26 (11–52)	231 (123–386)	54 (18–165)
Caste			
Scheduled caste	23 (10–48)	200(115–371)	34 (13–100)
Scheduled tribe	18 (9–41)	154 (75–240)	25 (10–67)
Backward class	25 (12–48)	229 (119–381)	54 (18–169)
General	37 (17–77)	261 (146–413)	88 (32–246)
Place of residence			
Rural	25 (11–50)	200 (111–346)	42 (16–122)
Urban	28 (12–61)	273 (154–457)	105 (29–295)
Catastrophic expenditure^a^ (%)	7%	60%	25%
Duration of treatment (in days)	3 (2–4)	5 (3–7)	4 (2–6)
Sale/borrowing of assets (%)	11	20	15
Medicines as a proportion of total cost	25	18	22
Indirect cost^b^ as a proportion of total cost	43	09	26

Weighted analysis done; cost estimates are given in terms of US dollars

The household monthly per capita consumption expenditure limits (in USD) for the five quintiles are as follows: first quintile (3–11), second quintile (12–24), third quintile (25–35), fourth quintile (36–52) and the fifth quintile (53–578)

*IQR* inter quartile range, *MPCE* monthly per capita expenditure

^a^Expenditure amounting to more than 10% of annual consumption expenditure is defined as catastrophic

^b^Indirect cost includes transport for patient and others, expenses on food, escort, lodging charges and others, etc

There was a clear inverse trend between proportion delivering in a public sector facility and income quintile. Public sector utilization by the poorest quintile (84%) was 2.5 times higher compared to the richest quintile (34%). Among the poorest quintile, expenditure in public sector hospitalizations was catastrophic in 13% of cases whereas it was 85% in private sector hospitalizations. In 15% of the hospitalizations, expenditure was sourced by borrowing or sale of physical assets (Table 2).

**Table 2:** Public health service utilization and impact of out-of-pocket expenditure on households from hospitalization due to childbirth across wealth quintiles in India, 2014

Wealth quintiles	Public health facility utilization	Catastrophic expenditure^a^ in public facility	Catastrophic expenditure in private facility	Borrowing/sale of assets
1st MPCE	84	13	85	16
2nd MPCE	72	06	75	16
3rd MPCE	63	04	60	16
4th MPCE	55	02	55	12
5th MPCE	34	01	44	8
Overall	64	07	60	15

Weighted analysis done; figures in the table represent percentages;

The household monthly per capita consumption expenditure limits (in USD) for the five quintiles are as follows: first quintile (3–11), second quintile (12–24), third quintile (25–35), fourth quintile (36–52) and the fifth quintile (53–578)

*MPCE* monthly per capita expenditure

^a^Expenditure amounting to more than 10% of annual consumption expenditure is defined as catastrophic

Logistic regression analysis shows that urban residence, poor wealth quintiles, residing in eastern and southern regions of India and delivery in a private hospital were significantly associated with catastrophic expenditure. However, women belonging to SC/ST and those belonging to Islam religion had lower chance of incurring catastrophic expenditure. (Table 3).

**Table 3:** Multivariate regression showing factors predicting catastrophic expenditure due to hospitalization from childbirth in India, 2014

Characteristics	Non-catastrophic expenditure, N (%)	Catastrophic expenditure, N (%)	Total, N	*P* value	Adjusted OR (CI)	P value
Place of residence				<0.001		
Rural	5488 (76)	1745 (24)	7233		1.0	
Urban	3809 (67)	1899 (33)	5708		1.2 (1.1–1.3)	0.003
Wealth quintiles				0.03		
1st MPCE	1893 (73)	700 (27)	2593		10.1 (8.3–12.2)	<0.001
2nd MPCE	2090 (72)	796 (28)	2886		4.7 (3.9–5.6)	<0.001
3rd MPCE	1993 (73)	737 (27)	2730		2.7 (2.3–3.2)	<0.001
4th MPCE	1668 (70)	712 (30)	2380		1.9 (1.6–2.2)	<0.001
5th MPCE	1655 (70)	699 (30)	2354		1.0	
Religion				0.19		
Hinduism	7162 (71)	2872 (29)	10,034		1.0	
Islam	1307 (73)	474 (27)	1781		0.8 (0.7–0.9)	0.01
Christianity	526 (73)	191 (27)	717		1.1 (0.9–1.4)	0.4
Others	302 (74)	107 (26)	409		1.4 (1.0–1.9)	0.02
Caste				<0.001		
SC/ST	3174 (81)	751 (19)	3925		0.6 (0.5–0.7)	<0.001
Other backward class	3604 (69)	1595 (31)	5199		0.8 (0.7–0.9)	0.002
General	2519 (66)	1298 (34)	3817		1.0	
Region				<0.001		
North	2471 (79)	673 (21)	3144		1.1 (0.8–1.3)	0.6
South	1643 (57)	1250 (43)	2893		2.8 (2.2–3.5)	<0.001
East	2647 (74)	932 (26)	3579		3.0 (2.3–3.8)	<0.001
West	1760 (73)	639 (27)	2399		1.0 (0.8–1.2)	0.8
Central	778 (84)	150 (16)	928		1.0	
Place of delivery						
Public	7391(92)	710 (8)	8101	<0.001	1.0	
Private	1908 (39)	2934 (61)	4842		38.6 (33.6–44.3)	<0.001

Weighted analysis done; expenditure amounting to more than 10% of annual consumption expenditure is defined as catastrophic; MPCE = monthly per capita expenditure; The household monthly per capita consumption expenditure limits (in USD) for the five quintiles are as follows: first quintile (3–11), second quintile (12–24), third quintile (25–35), fourth quintile (36–52) and the fifth quintile (53–578)

*SC* scheduled caste, *ST* scheduled tribe, *OR* odds ratio, *CI* confidence interval

In 64% of public sector hospitalizations, medicines had to be purchased out of pocket. Only 2.5% of the hospitalizations were insured either fully or to some extent. Among those who were insured, half of them belonged to the richest quintile whereas less than 10% belonged to the poorest quintile. (data not tabulated).

### Interstate variations

**Fig. 1:**
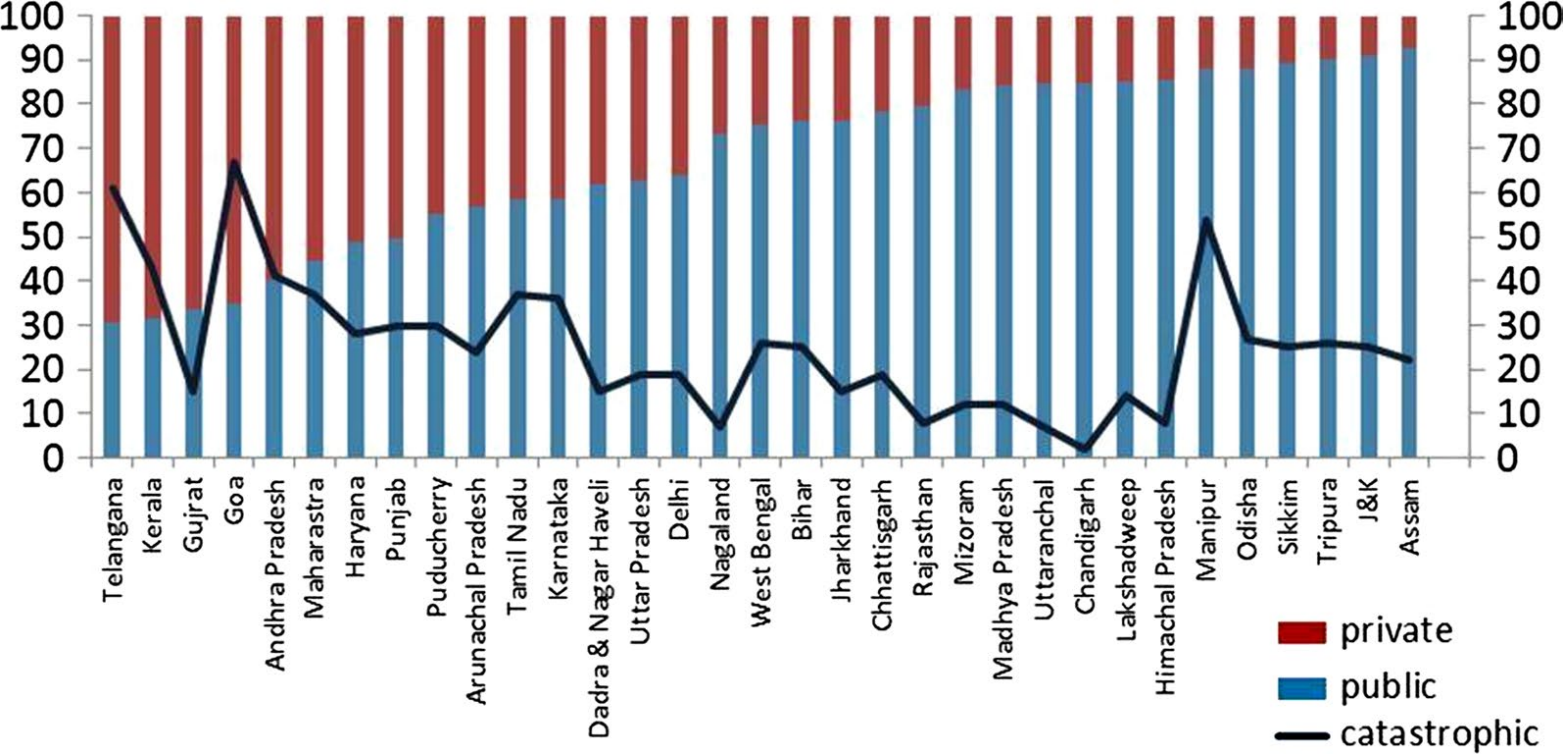
Public health service utilization and catastrophic expenditure from hospitalization due to childbirth in different states of India, 2014 Expenditure amounting to more than 10% of annual consumption expenditure is defined as catastrophic

**Fig. 2:**
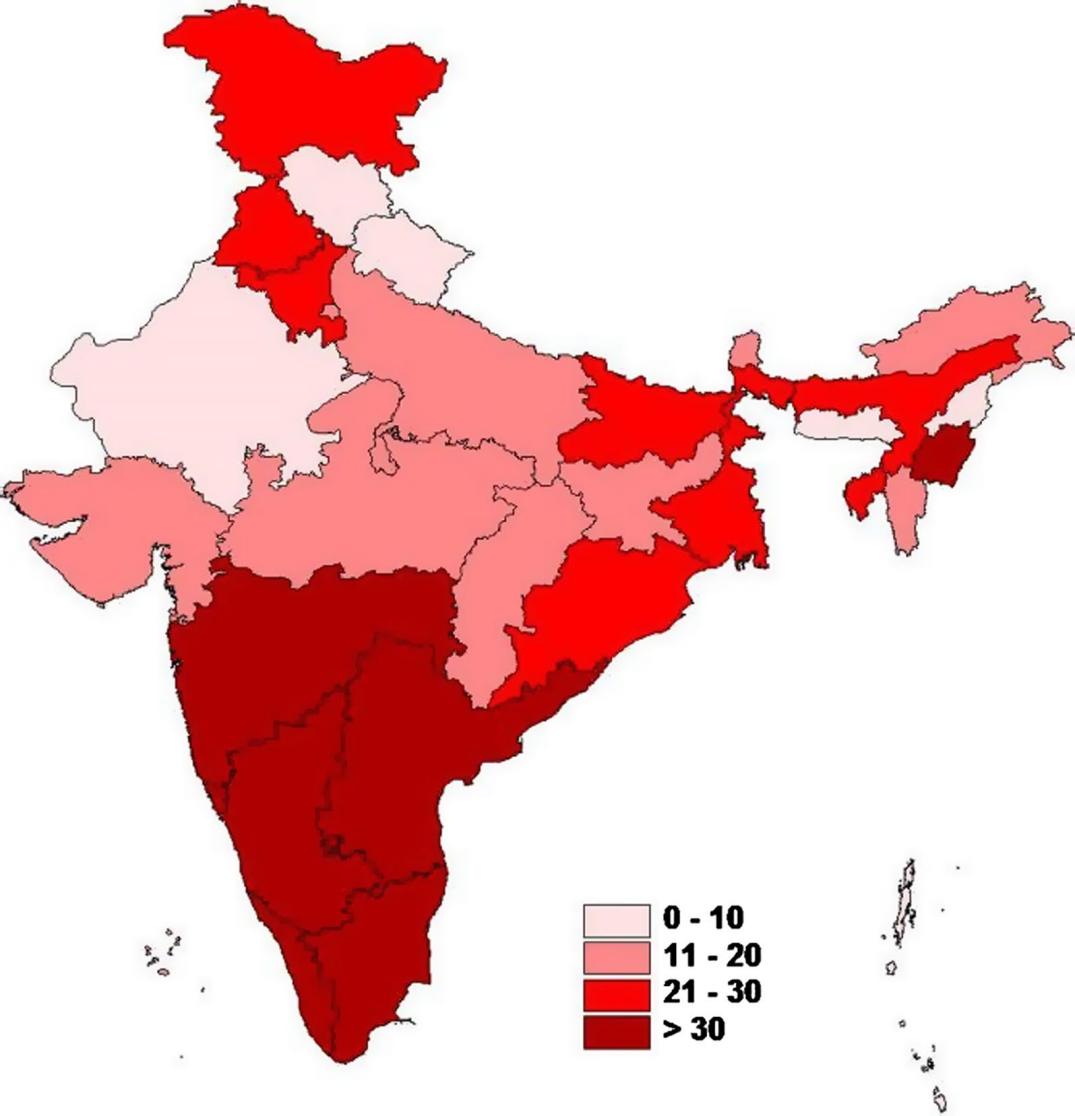
Map of India showing state-wise prevalence of catastrophic expenditure due to hospitalization from childbirth Shape file (.shp) for India state map was downloaded from http://www.diva-gis.org (open source)

The proportion of deliveries at private health facilities varied greatly among the states, with the lowest in Andaman and Nicobar islands, north eastern states (Assam, Meghalaya, Tripura, Meghalaya), J&K and Odisha, and highest in Daman and Diu, Telangana, Kerala, Gujarat, Goa and Andhra Pradesh. The proportion of deliveries resulting in catastrophic expenditure was maximum in Goa (67%) followed by Telangana (61%) whereas it was minimum in A&N islands and Chandigarh (2%). (Figs. 1, 2).

**Fig. 3:**
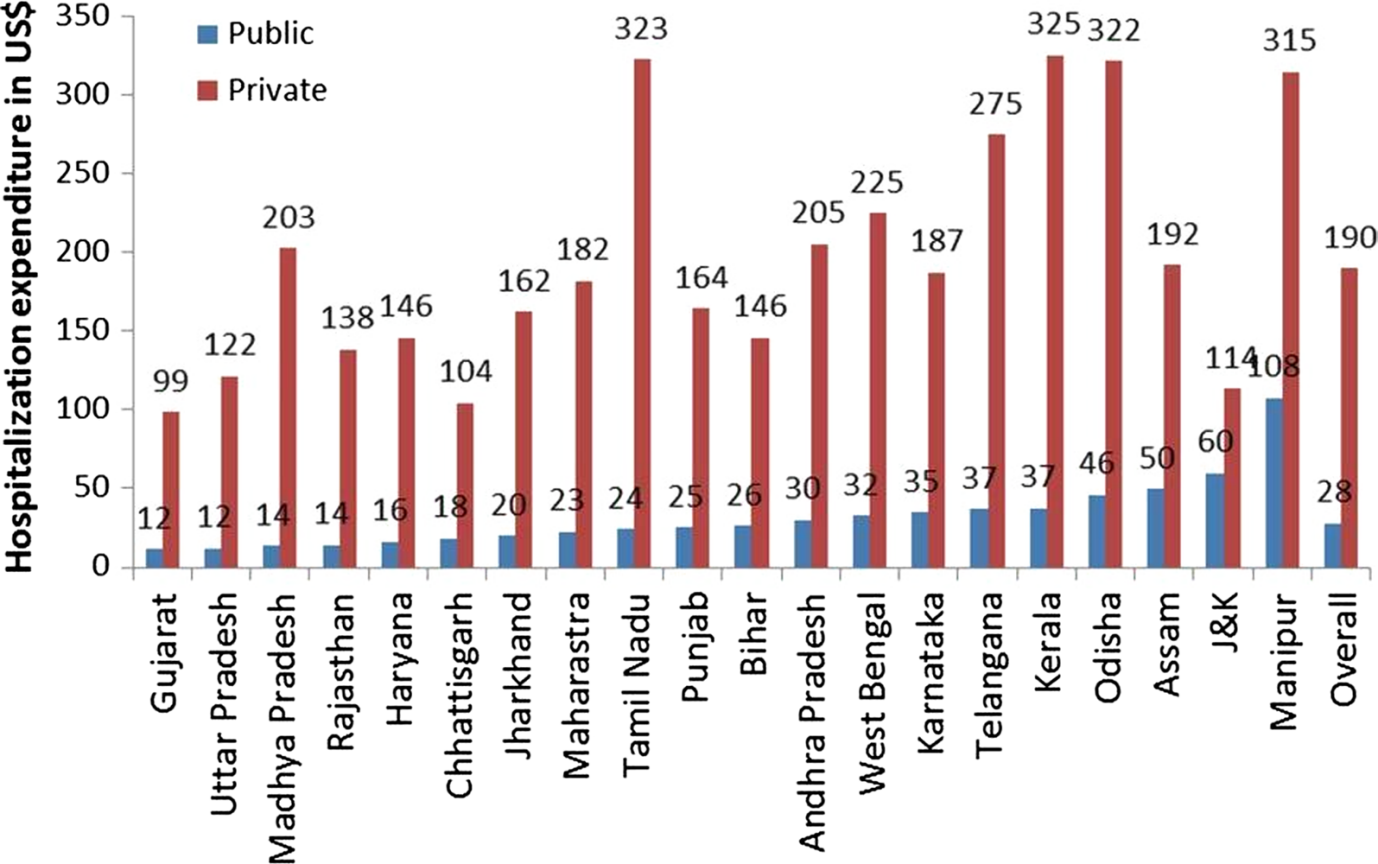
Hospitalization expenditure incurred by the poor* due to childbirth in public and private sector facilities in different states of India, 2014 Figures in the graph represent hospitalization expenditure in US Dollars (poor includes those belonging to the lowest two quintiles)

Overall, the poor spend US$28 and US$190 in public and private sector facilities respectively. Maximum public health spending by the poor was found in the state of Manipur (US$108) whereas it was minimal in Gujarat (US$12). Maximum private sector spending by the poor was in the states of Kerala (US$325), Tamil Nadu (US$323) and Odisha (US$322) whereas it was minimal in Gujarat (US$99). (Fig. 3).

## Discussion

Our analysis shows that there is still substantial OOP expenditure (US$26) incurred during childbirth even in post-NRHM era with wide inter-state variation. Hospitalization in a private sector facility led to a significantly higher prevalence of catastrophic expenditure than hospitalization in a public sector. Indirect costs constituted the largest share in the total expenditure in public sector hospitalizations.

Bonu et al. calculated maternal health care expenditure in the pre-NRHM period analysing a national survey done in 2004. The cost of delivery in a public facility was found to be US$25. This estimate needs to be interpreted with caution as it needs to be adjusted for inflation. In the same study, prevalence of catastrophic expenditure was found to be 21% in a public health facility which is much higher than the estimates reported in this study (7%) [16]. In another study by Mohanty et al. during the initial years of NRHM implementation (2004–08), the cost of delivery was found to be higher (US$39) in a public health facility (unadjusted for inflation) compared to the figures reported in this study (US$26) during 2014 [17]. Thus, there is a relative decline in the cost of delivery and prevalence of catastrophic expenditure in public health facilities during the post-NRHM period. Prinja et al., also showed that the OOP expenditures on delivery in the public sector decreased in the post-NRHM period by 73% in Haryana [21]. This decline might be attributed to the increased spending under the NRHM leading to better infrastructure and availability of drugs and supplies eventually resulting in reduced OOP expenditure on delivery care.

Similarly, other studies which have looked at the impact of cash-transfer schemes in the post-NRHM period such as the JSY and JSSK have found a substantial decrease in the OOP expenditures for delivery care [22, 23].

Prior to the launch of NRHM, utilization of public health facilities for delivery was poor. Bonu et al. (2004–05) and Mohanty et al. (2004–08) reported public sector utilization for delivery care to be 21 and 26% respectively which is way below compared to the post-NRHM period (64%) as reported in this study [16, 17]. This suggests that the NRHM interventions have resulted in a positive shift in the contribution of the public sector to overall institutional care at delivery.

However the results of this study show that the poor (lowest two quintiles) still spend a substantial amount on childbirth in public and private health facilities. This calls for strengthening or increasing the coverage of existing cash transfer schemes and low-cost private health care with effective pro-poor targeting. Studies have found that cash transfer schemes have a positive impact on the use of health services and health outcomes but they also emphasise the need for improved targeting of the poor [22, 23].

The results of this study confirm the important role that the private sector currently plays in the provision of health services for hospitalizations associated with childbirth. In the last decade there has been significant increase in private sector expenditure on healthcare. There is low public spending leading to inadequate public health infrastructure, shortage of human resources and other supplies. As a consequence of this neglect, there is a growing lack of trust in public health care due to poor access and service quality leading to rising dependence on private providers. Unregulated private sector delivery of healthcare is over-responsive to demand and exploitation. Thus, government needs to step into regulate the costs of services in private sector and at the same time utilize the private infrastructure to provide accessible and affordable maternal health care.

The full survey report states the following reasons for not availing government services due to any health issue: quality not satisfactory, long waiting, service not available or facility too far. Thus, the public sector needs to be strengthened in terms of quality of care, infrastructure and availability of services, providers and drugs in order to increase its access and utilization. Considering the inadequacy of the public sector, private providers need to be included and brought in through innovative public–private-partnership models. At the same time, costs of essential services in the private sector like maternity care should be regulated by negotiating price ceilings.

The expenditure incurred in public health facilities is mainly attributed to the indirect costs and unavailability of medicines. This is worrisome particularly for poor rural households where women visit facilities located in small towns or cities and often borrow money to cover transportation, food and accommodation costs [15, 16]. Another study conducted in India found poor availability of essential medicines in public health facilities forcing patients to purchase medicines from the private sector [24]. Thus, to increase access and affordability of health care, promotion of generic medicines, improved availability of medicines and subsidization of the poorest population quintiles in the public sector are required [25].

In states with high public sector utilization, the prevalence of catastrophic expenditure was low except in some north-eastern states, Odisha and J&K. This is probably due to weak public health infrastructure and poor availability of drugs and supplies in these low performing states. On the other hand, Gujarat, in spite of high private sector utilization had a very low prevalence of catastrophic expenditure. The government of Gujarat implemented the Chiranjeevi Scheme (CS), a PPP model to improve institutional delivery and reduce OOP expenditure in 2005. Studies suggest that the scheme is highly effective in reducing the OOP cost of delivery [26]. It also led to increase in institutional deliveries and better maternal and neonatal outcomes [27, 28]. Another study demonstrated that this scheme greatly improved the availability of comprehensive emergency obstetric care within reasonable travel distance [29]. This shows that it is possible to develop a large scale partnership with the private sector to provide skilled birth attendance and emergency obstetric care to poor women at a relatively low cost. The overall utilization of public maternity services and average expenditure incurred in the public facilities varied significantly across states in India. Given this wide variation, we need to understand the reasons behind this and design appropriate remedial measures at the state level.

The major strength in this study is the large recent nationally representative dataset used in this study. There are some methodological limitations to this study as well. Firstly, consumption expenditure in the survey does not differentiate between food and non-food expenditure. The WHO recommends a 40% cut-off level for non-food expenditure to estimate catastrophic expenditure [30]. We have used a threshold of 10% of annual consumption expenditure. However, this does not provide an accurate estimate of catastrophic expenditure since the expenditure on food as a proportion of total consumption expenditure is higher for poorer households. Hence the estimates of catastrophic expenditure might be an underestimate for the poorer income quintiles and an overestimate for higher income quintiles. Secondly, use of consumption expenditure as a proxy to income might overestimate the findings as well. Thirdly, indirect costs did not account for wage losses due to the illness which might have underestimated the impact of health care expenditure on the household.

## Conclusion

The study provides conclusive evidence of financial distress due to maternal expenditure, especially for the poorest quintile of the population which is a potential barrier to the use of maternal services. Therefore we strongly recommend cash transfer schemes with effective pro-poor targeting along with concurrent efforts to strengthen the public sector, regulate the private sector and engage private providers through innovative public–private partnership models. There is high contribution of drugs and indirect costs to the OOP in the public sector. We must ensure free essential generic drugs at all public health facilities. High indirect costs could be brought down by improving access to facilities by engaging private providers, free ambulance service or additional cash transfer.

## Abbreviations

OOP: out-of-pocket expenditure
JSSK: Janani Shishu Suraksha Karyakram (JSSK)
JSY: Janani Suraksha Yojana
NRHM: National Rural Health Mission
GDP: gross domestic product
MPCE: monthly per capita consumption expenditure
SDGs: sustainable development goals
NSSO: National Sample Survey Organisation
ECG: electro-cardiogram
